# The bastard grunt *Pomadasys incisus* (Bowdich, 1825) (Teleostei: Haemulidae) in Cyprus (eastern Mediterranean Sea) - a late arrival or just a neglected species?

**DOI:** 10.3897/BDJ.8.e58646

**Published:** 2020-11-23

**Authors:** Nikos Doumpas, Valentina Tanduo, Fabio Crocetta, Ioannis Giovos, Joachim Langeneck, Francesco Tiralongo, Periklis Kleitou

**Affiliations:** 1 iSea, Environmental Organization for the Preservation of Aquatic Ecosystems, Thessaloniki, Greece iSea, Environmental Organization for the Preservation of Aquatic Ecosystems Thessaloniki Greece; 2 Stazione Zoologica Anton Dohrn, Napoli, Italy Stazione Zoologica Anton Dohrn Napoli Italy; 3 Marine and Environmental Research (MER) Lab Ltd, Zygi, Cyprus Marine and Environmental Research (MER) Lab Ltd Zygi Cyprus; 4 Department of Biology, University of Pisa, Pisa, Italy Department of Biology, University of Pisa Pisa Italy; 5 Ente Fauna Marina Mediterranea, Avola, Italy Ente Fauna Marina Mediterranea Avola Italy; 6 Department of Biological, Geological and Environmental Sciences, University of Catania, Catania, Italy Department of Biological, Geological and Environmental Sciences, University of Catania Catania Italy

**Keywords:** citizen science, fishery, Perciformes, range expansion, thermophilous species

## Abstract

*Pomadasys incisus* is a thermophilous coastal subtropical fish species belonging to the family Haemulidae. Originally described from Gambia, this species is widely distributed in the Eastern Atlantic from Galicia to South Africa. It has also been recorded in the Mediterranean Sea since 1840, presumably expanding its distribution in the next decades, although the species could have been already present in the basin, but simply overlooked until the mid XIX century. In this study, we first record *P. incisus* from Cyprus (eastern Mediterranean Sea), based on two opportunistic observations obtained through a citizen-science project and review the distribution of this species in the Mediterranean Sea. The present sighting raises the question on whether this species is a late arrival in the country or its presence has just been neglected until now. Based on present data, the most likely hypothesis is the latter one, with *P. incisus* occurring in low densities and being overlooked due to the absence of field studies. Whatever is true, some intrinsic or extrinsic factors may have played a role in limiting its spread or wide establishment in the above-mentioned country.

## Introduction

The Mediterranean Sea is a biodiversity *hotspot* comprising approximately 17,000 marine species, including about 700 fish species (Coll et al. 2010, Psomadakis et al. 2012). Amongst them, the family Haemulidae Gill, 1885, includes five species belonging to the genus *Parapristipoma* Bleeker, 1873, namely *Parapristipoma octolineatum* (Valenciennes, 1833), to the genus *Plectorhinchus* Lacepède, 1801, namely *Plectorhinchus gaterinus* (Forsskål, 1775) and *Plectorhinchus mediterraneus* (Guichenot, 1850) and to the genus *Pomadasys* Lacepède, 1802, namely *Pomadasys incisus* (Bowdich, 1825) and *Pomadasys stridens* (Forsskål, 1775) (Fricke et al. 2020).

Within the Mediterranean, the latter two taxa have particularly received the attention of the Mediterranean scientific community. In fact, the striped piggy *P. stridens* is native to the Indo-Pacific and entered the Mediterranean Sea through the Suez Canal around 1888, when it was first observed in Port-Saïd (see Carus 1983, Bodilis et al. 2013), subsequently colonising the eastern Mediterranean basin up to Greece and being also known from Italy on the basis of a single record (Golani et al. 2002, Bilecenoglu et al. 2009, Kousteni et al. 2019, Servello et al. 2019, Bariche and Fricke 2020). Conversely, the bastard grunt *P. incisus* was originally described from Gambia and is widely distributed along the entire Eastern Atlantic coastline, from South Africa to Galicia (Palacky 1895, Gilchrist and Thompson 1908, Osorio 1909, Bauchot 1963, Bodilis et al. 2013, Bañón et al. 2014), including the Canary Islands (Vinciguerra 1883, Murray et al. 1912, Bianchi 1984), Madeira (Günther 1859, Andrade and Albuquerque 1935, Ribeiro et al. 2005) and Cape Verde Islands (Osorio 1909), but it has also been recorded in the Mediterranean Sea since the middle of the XIX century. In fact, despite its first record in the Mediterranean Sea dating back to 1840 (Guichenot 1850), the species could have been already present in the basin, but simply overlooked until the mid XIX century. Notwithstanding such doubts, the species was then recorded from Algerian, Tunisian, Spanish, French, Italian, Libyan, Egyptian, Israeli, Lebanese, Syrian, Turkish, Greek, Maltese and Croatian coasts (Bodilis et al. 2013, Fig. 1).

Such a broad distribution and dispersal capacity of *P. incisus* was indeed facilitated by its wide ecological requirements. In fact, *P. incisus* is a gonochoric species characterised by a fast growth and a moderately short-life (up to seven years) (Pajuelo et al. 2003a, Pajuelo et al. 2003b), inhabiting both marine and brackish waters from 10 to 100 metres depth (Kapiris et al. 2008), living on different types of substrates and environments, including hard and sandy bottom, seagrass meadows and even estuaries and lagoons (Bauchot 1963, Golani et al. 2006, Kapiris et al. 2008) and feeding on a wide range of invertebrates (Ben Tuvia and McKay 1984).

Despite the occurrences listed above, the published distribution of *P. incisus* in the Mediterranean basin still remains patchy (Bodilis et al. 2013) and its biology remains little investigated at an international level (Kapiris et al. 2008). Finally, only few studies reported high local abundances and records over the years, which suggests that some intrinsic or extrinsic factors may play a role in limiting its spread or a wide colonisation of the Mediterranean coastline (Bodilis et al. 2013). We hereby first report the presence of *P. incisus* in Cyprus on the basis of two opportunistic observations obtained through a citizen science project, thus filling a gap in the known Mediterranean distribution of this taxon.

## Material and methods

The present record falls within the framework of the iSea project “Is it alien to you? Share it!!!” (Giovos et al. 2019) that aims to monitor the expansion and establishment of marine alien species in the eastern Mediterranean and mainly in Greece and Cyprus, based on a citizen science approach. Furthermore, since the beginning of the project, a special focus has also been given to rare or overlooked species (Giovos et al. 2019), as in the present case.

**Figure 1.**
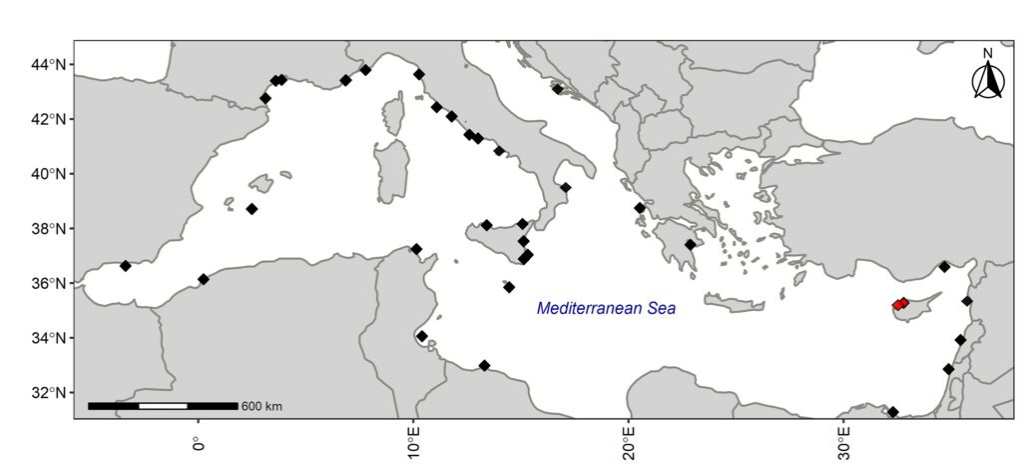
Map of known records of *Pomadasys incisus* in the Mediterranean Sea. Data after: Bilecenoglu et al. 2013, Bodilis et al. 2013, Karachle et al. 2016, Ali 2018, Tiralongo et al. 2019, Bariche and Fricke 2020, Tiralongo et al. 2020. Red dots indicate the present records from Cyprus (see below).

**Figure 2.**
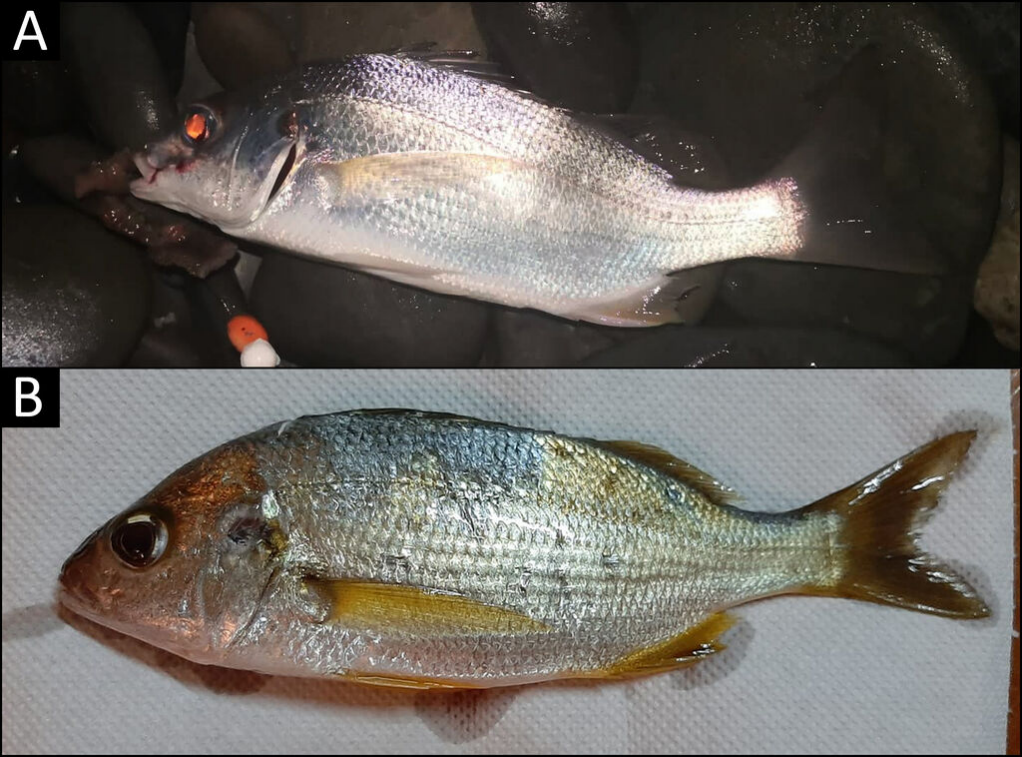
*Pomadasys incisus* from Cyprus. **A.** A specimen from Morfou Bay, total length around 17 cm (photographed by Marios Constantinides) (colours altered by the flash. **B.** A specimen from Limni beach, total length around 13 cm (photographed by Michael Antoniou).

On 30 September 2019, an experienced recreational fisherman from Cyprus captured four unidentified specimens (ranging 14–18 cm total length) north of Morfou Bay (35°11'15.4"N, 32°54'19.1"E), at 2 metres depth on a sandy bottom (Figs 1, 2A). On 20 May 2020, another recreational fisherman caught one specimen around 13 cm (total length) at Limni Beach (35°03'08.2"N, 32°26'36.4"E) on a sandy bottom (Figs 1, 2B). All specimens were caught during night, using a fishing rod from the shore and rag worms (family Nereididae Blainville, 1818) as bait. As the bastard grunt was unknown to them, both fishermen posted the photos of the specimens to the social media platform group mentioned above. Finally, a bibliographic research was carried in order to update and evaluate the distribution of this species in the Mediterranean Sea.

## Results

The specimens were identified as *P. incisus* due to their greyish colouration on the dorsum and a silvery-white colouration on the body side and belly, the dark spot on the upper part of the operculum and the fins orange or yellow, all characteristics that agree with the description of the species (Kaspiris 1970). Despite the specimens being eaten by the two fishermen, the features listed above make the distinction with the other Mediterranean *Pomadasys* species fairly easy, as *P. stridens* is longitudinally crossed by three yellowish or light brown bars, with the lower one extending from the eye to the caudal peduncle. Furthermore, the anal, pelvic and pectoral fins in *P. stridens* are usually whitish or light brown (McKay 2001). No other *Pomadasys* species is similar to the specimens caught in Cyprus with the sole exception of *Pomadasys auritus* (Cuvier, 1830). However, this is a Western Pacific species not yet recorded in the Mediterranean Sea. Moreover, *P. auritus* can be easily distinguished from *P. incisus* by different morphological and colour features, such as an elongated operculum that exceeds the level of the pectoral fin base (with *P. incisus* having a short operculum that does not reach the level of the pectoral fin base). Further, the small brownish rounded spots on the dorsal fin and the small blackish spots on the dorsal surface are present in *P. auritus* and absent in *P. incisus*. Finally, *P. incisus* has a dark spot on the posterior margin of the operculum, absent in *P. auritus* (*McKay 2001*).

## Discussion

The present study reports the presence of *P. incisus* in Cyprus, thus filling a gap in the known distribution of this species in the Mediterranean Sea. Bodilis et al. 2013 first suggested that *P. incisus* is a recent newcomer in the Mediterranean Sea and analysed its possible spread pattern while entering the Mediterranean Sea through the Straits of Gibraltar. In particular, two different dispersal pathways were mentioned: i) the first is along the coast of North Africa (Algeria and Tunisia), from where it may have subsequently spread in the entire basin; ii) the second is along the northern coast of the Mediterranean, thus reaching the coast of Spain and France in opposition to the main Mediterranean currents. Within the current climate change scenario, the expansion and establishment of this thermophilic species to the entire Mediterranean could be favoured by plasticity in life-history traits (egg size and quality being traded-off for higher egg numbers; lower lengths at maturity and alterations to spawning phenology) of different populations living in the basin (Villegas-Hernández et al. 2014). However, the absence of genetic studies investigating the connectivity of *P. incisus* populations in the Mediterranean Sea and along the African Coast makes it difficult to confirm or dispute such hypotheses. Thus, the species could also have been already present in the basin, but simply overlooked until the mid XIX century. This scenario is highly probable when taking into account that *P. incisus* was only formally described 15 years earlier and ichthyology was still in its early stages.

Notwithstanding these doubts, the present records immediately raise the question on the origin of the specimens recorded here. In fact, although *P. incisus* has been reported since the middle of the XIX century (Guichenot 1850) in the Mediterranean basin and should have already been present in Cyprus, the fact that the fish were unknown to both observers and the local fishing community, suggests at first glance that the species has made its appearance in the area only in the recent years. However, taking into account that the species had already been recorded also from the nearby coasts of Lebanon, Syria and Turkey ([Bibr B6108257][Bibr B6108206], Ali 2018, Bariche and Fricke 2020), it is most likely that it was already present in the area, but overlooked until now due to its local rarity. Indeed, this taxon can show very high abundances only where subtropical habitats and warmer environmental conditions prevail (e.g. Pajuelo et al. 2003a, Pajuelo et al. 2003b, Fehri-Bedoui and Gharbi 2008), characteristics that may not be present along the entire eastern Mediterranean. Moreover, the Cypriot coastline generally suffers from the absence of proper field studies and local monitoring in recent years has been often significantly complemented by opportunistic observations and citizen science (e.g. Kleitou et al. 2019, Paz-Sedano et al. 2019), tools that already proved to be particularly effective in reporting and monitoring rare native species or non-indigenous ones all over the Mediterranean Sea (e.g. Crocetta et al. 2017, Crocetta et al. 2020, Langeneck et al. 2019, Osca et al. 2019, Tanduo et al. 2020, Tiralongo et al. 2020).

Absence of published data regarding larval dispersal and/or genetic connectivity and local abundances of *P. incisus* in the Mediterranean Sea prevents us from resolving the question. Whatever is true, some intrinsic or extrinsic factors may have played a role in limiting its spread or wide establishment in the country and this may constitute an interesting baseline for future studies.
